# LuxR-Type Regulator RRP_6_ Positively Regulates the Biosynthesis of Plantaricin EF and Improves Its Production in *Lactiplantibacillus plantarum* 163

**DOI:** 10.3390/microorganisms13122780

**Published:** 2025-12-06

**Authors:** Yaxuan Liu, Siqi Liu, Zixian Li, Chuangen Huo, Guangli Wang, Xin Zeng, Bingyue Xin, Deyin Zhao

**Affiliations:** School of Life Science, Huaibei Normal University, Huaibei 235000, China; 13966096095@163.com (Y.L.); liusiqi7925@163.com (S.L.); 17364453053@163.com (Z.L.); douhua2026@163.com (C.H.); wanf-3344@163.com (G.W.); xzenghsd@163.com (X.Z.); xinbingyuex@163.com (B.X.)

**Keywords:** HPK_6_/RRP_6_, regulatory mechanism, plantaricin EF, improve production

## Abstract

The two-component system HPK_6_/RRP_6_ related to the *pln* locus of plantaricin biosynthesis was screened out. The overexpression of LuxR-type regulator RRP_6_ promoted the transcription of ABC transporter-related genes, thereby increasing plantaricin EF yield. Its yield in 163(*rrp_6_*) reached 16.01 mg/L, which was 1.20-fold that of the original strain. The regulatory mechanism indicated that RRP_6_ could bind to two sites of the *plnG1* promoter, promoting its transcription and translation, accelerating the secretion of plantaricin and auto-inducing peptide, and enhancing the extracellular plantaricin yield. Amino acids Q73, R144, T171, and Y175 play a crucial role in the binding of RRP_6_. Furthermore, potential regulatory compensation within the *Lactiplantibacillus plantarum* 163 genome may compensate for the negative effects after the deletion of *rrp_6_*. These results provide a novel strategy for increasing plantaricin EF yield, which facilitates its large-scale application as a natural and safe food preservative in agriculture and the food industry.

## 1. Introduction

Lactic Acid Bacteria (LAB) are widely recognized for their beneficial roles in human health, particularly in modulating immune function and the gut microbiome [1,2,3,4]. Among various metabolites produced by LAB, bacteriocins have become an effective bio-preservative and a potential alternative to traditional antibiotics and chemical additives in agriculture and the food industry [5,6,7].

Plantaricin, an important type of bacteriocin produced by LAB, exhibits significant diversity and exists in multiple classifications of LAB bacteriocins. An increasing number of studies indicate that plantaricin can effectively inactivate foodborne pathogenic microorganisms to mitigate contamination in meat, seafood, milk, vegetables, and fruits [8,9]. Plantaricin not only extends shelf life but also serves as a potential alternative to chemical preservatives, highlighting its considerable application value in food preservation [10,11,12]. However, the limited production of plantaricin in wild bacterial strains poses challenges for large-scale production and application in the field of food safety [13].

It has been reported that plantaricin biosynthesis is regulated by both the quorum-sensing system (QSS) and the two-component system (TCS) [14]. The biosynthesis of bacteriocins in LAB is regulated by the relevant QSS in the gene cluster where the bacteriocin synthesis gene is located, and the auto-inducing peptide (AIP) mediated quorum sensing plays a key role in the biosynthesis of plantaricin in general [15,16]. Meanwhile, the biosynthesis of bacteriocins is also regulated by the TCS, such as sakacin A, whose biosynthesis is regulated by the TCS SapK/SapR in *Lactobacillus sakei* Lb70 strain [17]. The biosynthesis of carnobactin is regulated by the TCS CbnK/CbnR in *Carnobacterium piscicola* LV17A strain [18].

Typically, a TCS consists of a membrane-associated sensor histidine kinase (HK) and a cytoplasmic response regulator (RR). Upon perceiving specific environmental signals, the HK autophosphorylates and then transfers the phosphate group to its cognate RR. The phosphorylated RR subsequently undergoes a conformational change, enabling it to bind target sequences and modulate the transcription of downstream genes, thereby allowing the bacterium to adapt to changing conditions [19].

Plantaricin EF, a class of IIb bacteriocin produced by *Lactiplantibacillus plantarum* 163 (*L. plantarum* 163), was identified in our previous studies, which exhibits broad-spectrum and high-efficiency antimicrobial activity, but its yield is relatively low and its regulatory mechanism is rather complex [20]. In order to study its regulatory mechanism and improve the yield of plantaricin, the genome, transcriptome and proteome of *L. plantarum* 163 were determined in the previous stage of this study [20]. Through multi-omics analysis, it was determined that there is a certain correlation between the TCS HPK_6_/RRP_6_ and the *pln* locus that regulates the biosynthesis of plantaricin. Furthermore, the overexpression of regulatory factor RRP_6_ was found to improve the yield of plantaricin EF. Nevertheless, the regulatory mechanism by which the HPK_6_/RRP_6_ regulates the biosynthesis of plantaricin remains unclear, including the pathway through which RRP_6_ regulates plantaricin biosynthesis, the gene sequences bound by RRP_6_, and its binding mode. In this study, the regulatory mechanism of HPK_6_/RRP_6_ on plantaricin biosynthesis was investigated in *L. plantarum* 163. The plantaricin EF yield and expression levels of related genes were determined when the gene *rrp_6_* was overexpressed and knocked out, respectively. The nucleic acid sequences that are specifically bound by RRP_6_ protein were identified. In addition, the binding sites, affinity, and mode of interaction with the binding sequence for RRP_6_ protein were investigated. By combining the previous omics data and the current research results, the molecular mechanism by which RRP_6_ regulates its biosynthesis was elucidated, facilitating the construction of high-yield plantaricin strains, increasing the yield of plantaricin, and promoting the industrial production and application of plantaricin. 

## 2. Materials and Methods

### 2.1. Strains and Culture Medium

*L. plantarum* 163 was isolated from traditionally fermented cabbage in Guizhou, China, and preserved in the China General Microbial Culture Collection Center (No. 8224). *E. coli* DH5α and BL21(DE3) were preserved in our laboratory. *L. plantarum* 163 was cultured in de Man, Rogosa, and Sharpe (MRS) medium at 37 °C, while *E. coli* DH5α and BL21(DE3) were cultured in Luria–Bertani (LB) medium. The plasmids pET30a and pMG36e served as vectors for the target genes. *E. coli* DH5α and BL21(DE3) were used as hosts for plasmid replication and expression, respectively. Kanamycin or erythromycin (Solarbio, Beijing, China) was added to the medium based on the resistance gene present on the plasmid. The relevant plasmids and strains used in this study are shown in Table S1.

### 2.2. Construction of Overexpression and Heteroexpression Strains

Plasmids pMG36e and pET30a were used as overexpression and heterologous expression vectors for the *rrp_6_* gene, respectively. Primers e-F/e-R and e-*rrp_6_*F/e-*rrp_6_*R were employed to generate linearized forms of plasmids pMG36e and the *rrp_6_* gene, respectively. The circular plasmid pMG36e-*rrp_6_* was constructed using a recombinant enzyme provided by ClonExpress II One Step Cloning Kit (Vazyme, Nanjing, China), and transferred into *L. plantarum* 163 via electroporation [21]. Meanwhile, the strain *L. plantarum* 163 containing the empty plasmid pMG36e was used as the control group. Similarly, primers a-F/a-R and a-*rrp_6_*F/a-*rrp_6_*R were used to linearize the plasmid pET30a along with the *rrp_6_* gene. These fragments were assembled using recombinant enzymes to form a circular plasmid pET30a-*rrp_6_* which was then introduced into *E. coli* BL21(DE3) through chemical transformation [22]. The strain *E. coli* BL21(DE3) containing the empty plasmid pET30a was used as the control group. The sequences of all recombinant plasmids were verified through nucleotide sequencing analysis. The relevant primers used in this study are shown in Table S2.

### 2.3. Screening the Regulatory Factors Related to the pln Locus of Plantaricin Biosynthesis

The whole genome, transcriptome and proteome of *L. plantarum* 163 were sequenced at the Beijing Genomic Institute (BGI, Shenzhen, China). Then, the whole genome, transcriptome and proteome data were analyzed using the BGI Interactive Cloud Platform (https://report.bgi.com/ps/login/login.html, accessed on 24 April 2025). Based on the whole genome analysis of *L. plantarum* 163, the *pln* locus of plantaricin biosynthesis was screened out. Through the joint analysis of the transcriptome and proteome of *L. plantarum* 163, genes with significant differences (fold change > 2 and Q-value ≤ 0.001 for transcriptional levels, and fold change > 1.5 and *p*-value < 0.05 for protein levels) at both the transcriptional and protein levels were screened out. Subsequently, in order to find the proteins related to the plantaricin biosynthesis *pln* locus, the protein–protein interaction analysis was performed on the BGI Interactive Cloud Platform. Meanwhile, the STRING database (https://string-db.org/, accessed on 7 May 2025) was adopted to further analyze the protein–protein interaction results. Additionally, the whole genome and transcriptome data were submitted to the Sequence Read Archive database of the National Center of Biotechnology Information (accession no. SRR12825168 and SRR12831476). The proteome data were uploaded to the National Protein Science Center (Beijing), China (accession no. PXD022981).

### 2.4. Effects of rrp_6_ Gene Overexpression on Plantaricin EF Production

The activated seeds of *L. plantarum* 163 and *L. plantarum* 163(*rrp_6_*) were cultured in MRS medium respectively at 37 °C for 52 h. Samples were collected every 4 h to assess the cell density, which was measured as the OD_600_ of the fermentation broth. Additionally, the culture medium at intervals of 12, 24, 36, and 48 h was taken out to harvest the cell-free supernatant (CFS) by centrifugation. The CFS was filtered with a 0.22 μm filter membrane. The plantaricin EF production in CFS was quantified utilizing reversed-phase high-performance liquid chromatography (RP-HPLC, UltiMate 3000, Thermo Scientific, Waltham, MA, USA) following the method previously described in our research [23]. The wild-type strain *L. plantarum* 163 was used as the control. Samples from both strains were prepared in triplicate, and the experiment was independently conducted with three parallel tests.

### 2.5. Effects of rrp_6_ Gene Overexpression on the Transcriptional Levels of Related Genes in the pln Locus

The Trizol method was employed to extract total RNA from the cell pellet collected by centrifugation as described in Section 2.4. The cDNA was synthesized using the HiScript 1st Strand cDNA Synthesis Kit (Vazyme, Nanjing, China). The qPCR system consisted of a total volume of 20 μL, 1.0 μL cDNA, 8.2 μL sterile ultrapure water, 10.0 μL Hieff qPCR SYBR Green Master Mix (Yeasen, Shanghai, China), 0.4 μL reverse primer, and 0.4 μL forward primer. Sterile ultrapure water served as a negative control in place of cDNA samples. The gene encoding 16S rRNA was utilized as a reference gene throughout this study. Wild-type strain *L. plantarum* 163 was used as the control. The transcription levels of target genes were analyzed using a StepOnePlus Real-Time PCR system (Veriti, Applied Biosystems, Waltham, MA, USA). Relative transcriptional levels of target genes were calculated using the 2^(−ΔΔCt)^ method [24,25]. All reactions were performed in sextuplicate to ensure result reliability and the experiment was independently conducted with three parallel tests. The qPCR primers used in this experiment are shown in Table S3.

### 2.6. Heterologous Expression and Purification of RRP_6_ Protein

*E. coli* BL21(DE3) containing pET30a-*rrp_6_* was used to prepare the seed solution. It was cultured at 37 °C and 180 rpm for 12 h in a shake flask with LB medium (100 mL). Subsequently, 1% of this inoculum was transferred to a shake flask containing fresh LB medium supplemented with kanamycin and cultured under the same conditions of 37 °C and 180 rpm. When the optical density at 600 nm wavelength reached 0.6–0.8, Isopropyl-β-D-Thiogalactopyranoside (IPTG; Macklin, Shanghai, China) was added to the culture at a final concentration of 100 μg/mL. The culture was then incubated at 16 °C and 180 rpm for an additional 20 h. After centrifugation at 8000 rpm for 5 min, the cell pellet was resuspended in a buffer (50 mM Tris-HCl, pH 7.1, 300 mM NaCl, 8% glycerol, Beyotime Biotech, Shanghai, China), and subjected to ultrasonication treatment at 0 °C for 15 min. Following another round of centrifugation (10,000 rpm, 10 min, 4 °C), the supernatant was added to a Ni-NTA column to facilitate the binding of RRP_6_ protein with Ni-NTA resin (Beyotime Biotech, Shanghai, China). Then, weakly bound proteins were removed with elution buffer (50 mM Tris-HCl, pH 7.1, 300 mM NaCl, 8% glycerol, 25 mM imidazole, Beyotime Biotech, Shanghai, China). Subsequently, RRP_6_ protein labeled with His-tag was eluted from the column using elution buffers containing imidazole at concentrations of 50, 100, and 200 mM. Then, the imidazole was removed from the RRP_6_ protein solution using a Millipore protein ultrafiltration tube (10 kDa; Millipore, Burlington, MA, USA). The eluent underwent further analysis via SDS-PAGE. *E. coli* BL21(DE3) carrying pET30a served as a control. The experiment was independently conducted in three parallel tests.

### 2.7. In Vitro Binding of RRP_6_ Protein to Sequence of plnG1 Promoter

For Electrophoretic Mobility Shift Assay (EMSA), slight modifications were made based on the method of Ueharu et al. [26]. Briefly, RRP_6_ protein was incubated with 50 ng of a FAM-labeled probe (Sangon Biotech, Shanghai, China) in a binding buffer (10 mM HEPES, pH 7.9, 0.1 mM spermidine, 0.4 mM MgCl_2_, 50 mM NaCl, 0.1 mM ZnCl_2_, 0.4 mM DTT, 8% glycerol; Beyotime Biotech, Shanghai, China) at 37 °C for 30 min. For the specific binding reaction, the FAM-labeled probes were replaced with 5 μg of unlabeled probes in the reaction mixture, which was further incubated at 37 °C for 30 min. Subsequently, the FAM-labeled probes (50 ng) were added to the reaction mixture and incubated at 37 °C for another 30 min. Following these steps, a polyacrylamide gel with a concentration of 4% was employed to conduct electrophoresis for 2 h at a voltage setting of 80 V. Finally, the fluorescence imaging instrument (Typhoon 9410, GE Healthcare, Chicago, IL, USA) was used to analyze the changes in electrophoretic mobility.

DNase I footprinting was performed following the method described by Chang et al. with minor modifications [27]. Varying concentrations (40 μL) of RRP_6_ protein and the probes (350 ng) were mixed and incubated at 25 °C for 30 min. Next, a 10 μL solution containing freshly prepared CaCl_2_ (100 nM, Kemiou Chemical Reagent Co., Ltd., Tianjin, China) and DNase I (0.015 U, Beyotime Biotech, Shanghai, China) was added to the mixture, followed by incubation in a metal bath at 37 °C for 1 min. Subsequently, 140 μL of DNase I termination solution was added to terminate the above reaction. Then, the samples were extracted with phenol/chloroform and subsequently precipitated with ethanol. Finally, the pellet was dissolved in ultrapure water (30 μL), and GeneScan-LIZ600 size standards (Applied Biosystems, Waltham, MA, USA) were used to analyze the results.

### 2.8. Molecular Docking

The interaction between RRP_6_ protein and the *plnG1* promoter sequence was considered through computational simulation. The structural models of RRP_6_ protein and binding sequences were obtained through homology modeling and Discover Studio 4.5 software, respectively. Molecular docking analysis was performed via ZDOCK 3.0.3 software, which employs fast Fourier transform technology for spatial searching, allowing for rapid identification of potential binding modes between two molecules in three-dimensional space [28]. Furthermore, ZDOCK utilizes scoring functions, dynamically adjusts the orientation of the input protein, and incorporates experimental binding data along with bioinformatics approaches, demonstrating high efficiency and accuracy throughout the molecular docking process [29,30]. For this study, the highest-scoring conformation was selected from a total of 100 successful docking results.

### 2.9. Site-Directed Mutagenesis of Key Amino Acids

According to the results of molecular docking, mutations were introduced at the two binding sites. Amino acids Q73, R144, T171, and Y175 were successively mutated into alanine. Site-specific mutant strains were constructed via PCR. The PCR products were combined with the linearized plasmid pET30a to construct circular plasmids pET30a-*rrp_6_*-∆Q73, pET30a-*rrp_6_*-∆R144, pET30a-*rrp_6_*-∆T171, and pET30a-*rrp_6_*-∆Y175, which were further transformed into *E. coli* BL21(DE3) respectively by the chemical transformation method [22]. Subsequently, single colonies were selected and inoculated into LB liquid medium. These single colonies were further sequenced by Sangon Bioengineering Co., Ltd. (Shanghai, China) to confirm the mutation sequence.

### 2.10. EMSA of Mutated Protein and Binding Sites

Four mutant proteins (RRP_6_-∆Q73, RRP_6_-∆R144, RRP_6_-∆T171, and RRP_6_-∆Y175) were expressed and purified in *E. coli* BL21(DE3). Methods of heterologous expression and purification were described in Section 2.6 above. The EMSA method employed was as described in Section 2.7 above. FAM-labeled probes and four mutant proteins were mixed in a binding buffer respectively, followed by incubation at 37 °C. Then, the samples were analyzed by EMSA, and the changes in mobility were observed through fluorescence imaging.

### 2.11. Gene Knockout of rrp_6_

*L. plantarum* 163(∆*rrp_6_*) was constructed based on the method described by Fuhren et al. with slight modifications [31]. Firstly, the sequences of the upstream and downstream homologous arms of the *rrp_6_* gene and the chloramphenicol resistance gene *cat* were obtained through PCR amplification in vitro. These three sequences were sequentially connected into a single fragment using an overlapping extension PCR method, wherein the resistance gene was positioned centrally, flanked by the upstream and downstream homologous arms of *rrp_6_* at both ends. The resulting fragment was subsequently inserted into plasmid pNZ5319 via one-step cloning to construct the knockout plasmid pNZ5319-*rrp_6_*, which was further electroporated into competent cells of *L. plantarum* 163. Then, 900 μL of liquid MRS medium was rapidly added to the electroporation cuvette and gently mixed. 1 mL of this mixture was transferred to 1.5 mL centrifuge tubes and incubated at 37 °C for 3 h. Subsequently, 100 μL of bacterial suspension was spread onto solid MRS medium plates containing 10 μg/mL chloramphenicol and cultured at 37 °C for 48 h. Single colonies were selected using toothpicks and inoculated into new centrifuge tubes containing 1 mL liquid MRS medium for subculturing.

After several generations, the bacterial cultures were diluted and plated onto solid MRS medium containing chloramphenicol. Single colonies were selected for further analysis. PCR was performed with primers flanking the *rrp_6_* homologous arm to verify the successful integration of the chloramphenicol resistance gene into the genome of *L. plantarum* 163. Meanwhile, specific primers at both ends of the *rrp_6_* gene were employed to confirm the loss of plasmid pNZ5319. The correct amplification of PCR products with primers surrounding both homologous arms of the *rrp_6_* gene, coupled with the failure to detect the *rrp_6_* gene itself, indicated that free plasmid pNZ5319 had been successfully eliminated. Furthermore, it was confirmed that the chloramphenicol resistance gene *cat* had replaced the *rrp_6_* gene in the genome of *L. plantarum* 163, thereby completing the construction of a *rrp_6_* knockout strain.

### 2.12. Effects of rrp_6_ Gene Knockout on the Growth Curve of L. plantarum 163 and the Plantaricin Yield

The activated wild-type *L. plantarum* 163 and knockout strains *L. plantarum* 163(∆*rrp_6_*) were introduced into MRS medium respectively and cultured at 37 °C for 48 h. The optical density of fermentation broth at 600 nm was measured to assess bacterial growth. For the yield determination of plantaricin EF, the method was slightly modified according to that of Zhao et al. [20]. The activated seeds of *L. plantarum* 163 and *L. plantarum* 163(∆*rrp_6_*) were inoculated into fresh MRS medium respectively and incubated at 37 °C. At time points of 12, 24, 36, and 48 h, the medium was centrifuged to collect the CFS and the plantaricin EF yield in the CFS was determined using RP-HPLC. All experiments were independently conducted three times, and three parallel tests were set up for each experiment.

### 2.13. Effects of rrp_6_ Gene Knockout on the Transcription Level of Relevant Genes

The methods used in this section were the same as those described in Section 2.5. Following the completion of RNA extraction, cDNA synthesis and qPCR detection were performed. The qPCR reaction was conducted in a 20 μL system, comprising 10 μL of SYBR Green Master Mix, 0.4 μL each of upstream and downstream primers, 1 μL of cDNA template, and 8.2 μL of sterile ultrapure water. The gene encoding 16S rRNA was utilized as a reference gene. Wild-type strain *L. plantarum* 163 was used as the control. The transcription levels of target genes were analyzed using a StepOnePlus Real-Time PCR system (Veriti, Applied Biosystems, Waltham, MA, USA). Relative quantification values for target genes were calculated employing the 2^−ΔΔCt^ method [24,25]. All reactions were performed in sextuplicate to ensure result reliability and the experiment was independently conducted with three parallel tests.

### 2.14. Statistical Analysis

Each experiment in this study was independently conducted with three parallel tests. The data presented in figures and tables were analyzed using SPSS software (version 27.0, IBM, Armonk, NY, USA), with results expressed as mean ± standard deviation (Mean ± SD). The significance of differences between groups was assessed using Duncan’s multiple range test. Statistically significant differences were marked as *p* < 0.05. The different lowercase letters indicate a significant difference in the same group.

## 3. Results

### 3.1. TCS HPK_6_/RRP_6_ Associated with the pln Locus of Plantaricin Biosynthesis

Through multi-omics joint analysis, a plantaricin biosynthesis gene cluster *pln* locus was found. The *pln* locus contains six operons, namely *plnorf3orf5orfZ3*, *plnKLR*, *plnABD*, *plnEFI*, *plnG1G2HSTUVW*, and *plnXY*. The functions of the related genes in these operons were shown in Table 1. As can be seen from Table 1, these genes are primarily involved in biosynthesis (*plnorf3*, *plnK*, *plnE*, *plnF*), immunity (*plnorf5*, *plnL*, *plnI*), regulation (*plnA*, *plnB*, *plnD*), and secretion (*plnG1*, *plnG2*, *plnH*, *plnS*, *plnT*, *plnU*, *plnV*, *plnW*) of plantaricin. Through protein–protein interaction analysis, HPK_6_/RRP_6_ was found to be correlated with the *pln* locus (Figures S1 and S2).

### 3.2. Effects of rrp_6_ Overexpression on the Growth of L. plantarum 163 and Plantaricin EF Production

**Figure 1:**
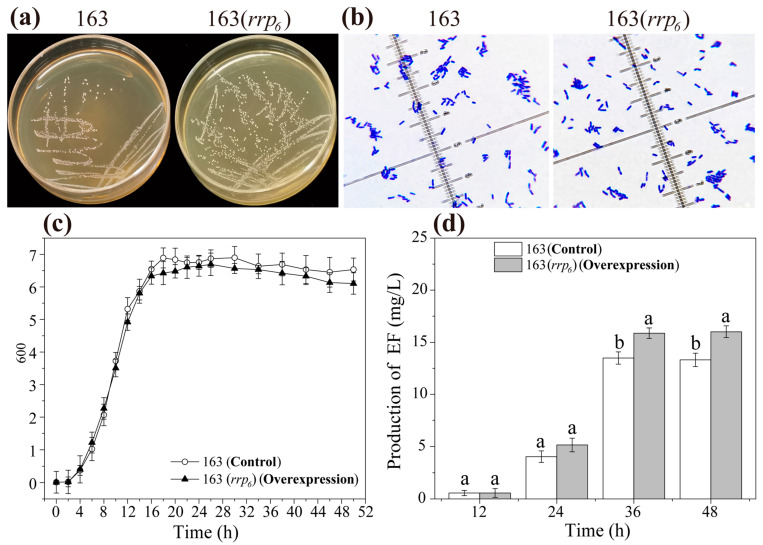
Effects of *rrp_6_* overexpression on the growth of *L. plantarum* 163 and plantaricin EF production. (**a**): Growth status; (**b**): Gram staining; (**c**): Growth curves; (**d**): Plantaricin EF production. 163: *L. plantarum* 163; 163(*rrp_6_*): *rrp_6_* gene overexpression in *L. plantarum* 163. The different lowercase letters indicate significant differences in the same group (*p* < 0.05). Mean ± SD, *n* = 3, technical replicates.

The growth characteristics and Gram staining properties of both the wild-type strain and the *rrp_6_* overexpression strain were found to be consistent (Figure 1a,b). The colonies produced by each strain were round with raised surfaces (Figure 1a). The results of Gram staining revealed a blue-purple coloration, and all cells exhibited a rod-shaped morphology (Figure 1b). As can be seen from Figure 1c and Figure S3, the growth curves of the *L. plantarum* 163 strain and *L. plantarum* 163(*rrp_6_*) strain were largely comparable. This observation indicates that the overexpression of the *rrp_6_* gene did not significantly impact the fundamental cellular metabolism or cell cycle of *L. plantarum* 163. Figure 1d and Figure S4 demonstrated that the production of plantaricin EF in the *L. plantarum* 163(*rrp_6_*) strain exhibited an increasing trend between 12 and 36 h, stabilizing from 36 to 48 h. By 48 h, the production in *L. plantarum* 163(*rrp_6_*) was 16.01 ± 0.55 mg/L, which was approximately 1.20-fold compared to the control group (*p* < 0.05). These results suggested that RRP_6_ specifically enhanced plantaricin EF biosynthesis without compromising cellular growth.

### 3.3. Effects of rrp_6_ Overexpression on Transcription Levels of pln Locus Associated Genes

**Figure 2:**
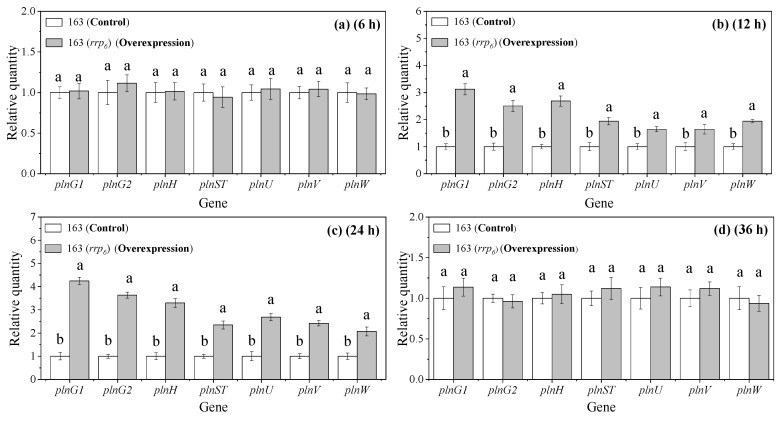
Effects of *rrp_6_* gene overexpression on the related genes of the *pln* locus. (**a**) Transcription levels at 6 h; (**b**) Transcription levels at 12 h; (**c**) Transcription levels at 24 h; (**d**) Transcription levels at 36 h. 163: *L. plantarum* 163; 163(*rrp_6_*): *rrp_6_* gene overexpression in *L. plantarum* 163. The different lowercase letters indicate significant difference in the same group (*p* < 0.05). Mean ± SD, *n* = 6, technical replicates.

As illustrated in Figure 2, the overexpression of *rrp_6_* in *L. plantarum* 163 resulted in a transcriptional pattern for the *plnG1*, *plnG2*, *plnH*, *plnST*, *plnU*, *plnV*, and *plnW* genes that initially increased and subsequently decreased. The transcription levels of these genes peaked between 12 h and 24 h, followed by a decline to levels comparable to those of the control by 36 h. At 24 h, the transcription levels of these genes within the overexpression were approximately 4.25-, 3.64-, 3.30-, 2.35-, 2.69-, 2.42- and 2.07-fold higher than those of the control group, respectively (*p* < 0.05). This indicated that the overexpression of the *rrp_6_* gene could significantly enhance the transcription levels of related regulatory genes.

### 3.4. Identification of plnG1 Promoter Binding Sequence to RRP_6_ Protein

**Figure 3:**
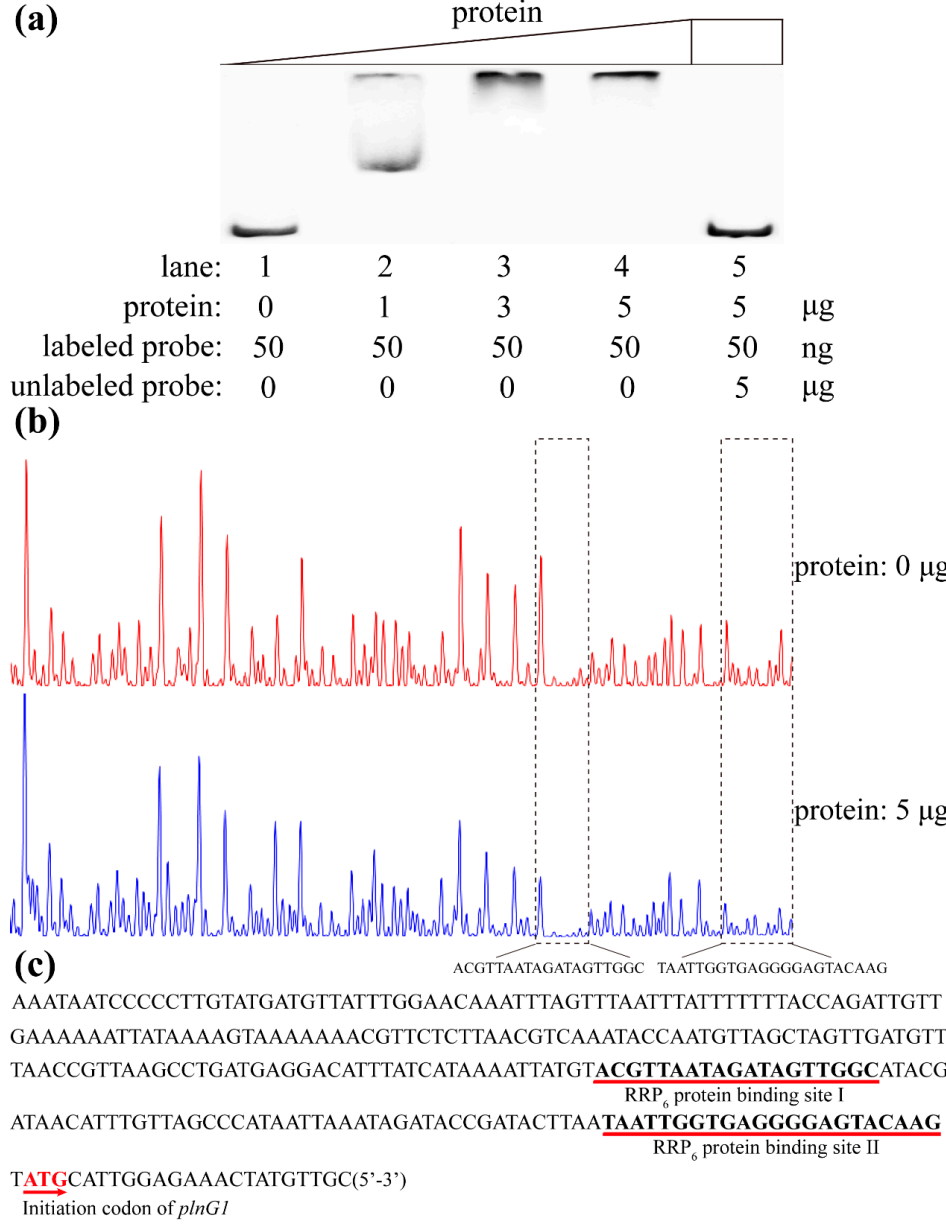
Analysis of EMSA and DNase I Footprinting assays of protein RRP_6_ and *plnG1* promoter. (**a**): Analysis of EMSA; (**b**,**c**): Analysis of DNase I footprinting.

The RRP_6_ protein with a molecular weight of 22.9 kDa was expressed in *E. coli* BL21(DE3) and purified by Ni-NTA column (Figures S5 and S6). The results of EMSA indicated that the RRP_6_ protein could bind to the *plnG1* promoter sequence (266 bp probe) (Figure 3a). As the concentration of RRP_6_ protein increased, there was a gradual decrease in band migration distance until it ultimately disappeared. At a protein concentration of 5 μg (lane 4), no band migration was observed, with all probes remaining within the well. This demonstrates that RRP_6_ protein can bind to the promoter sequence of *plnG1*. Meanwhile, the band migration distance in Lane 5 was almost the same as that in Lane 1, indicating that when sufficient unlabeled probes were added, RRP_6_ protein could bind to the unlabeled probes. Subsequently, when FAM-labeled probes were added, RRP_6_ protein no longer bound to the FAM-labeled probes. This result indicated that RRP_6_ protein binds specifically to the promoter sequence of *plnG1*. In addition, the DNase I footprinting assays showed similar results (Figure 3b,c). RRP_6_ protein could specifically bind to the promoter sequence of *plnG1*, and there were two specific binding sequences, which were Site I (ACGTTAATAGATAGTTGGC, 5′-3′, 19 bp) and Site II (TAATTGGTGAGGGGAGTACAAG, 5′-3′, 22 bp), respectively (Figure 3c).

### 3.5. Binding of RRP_6_ Protein to Two Sequences

**Figure 4:**
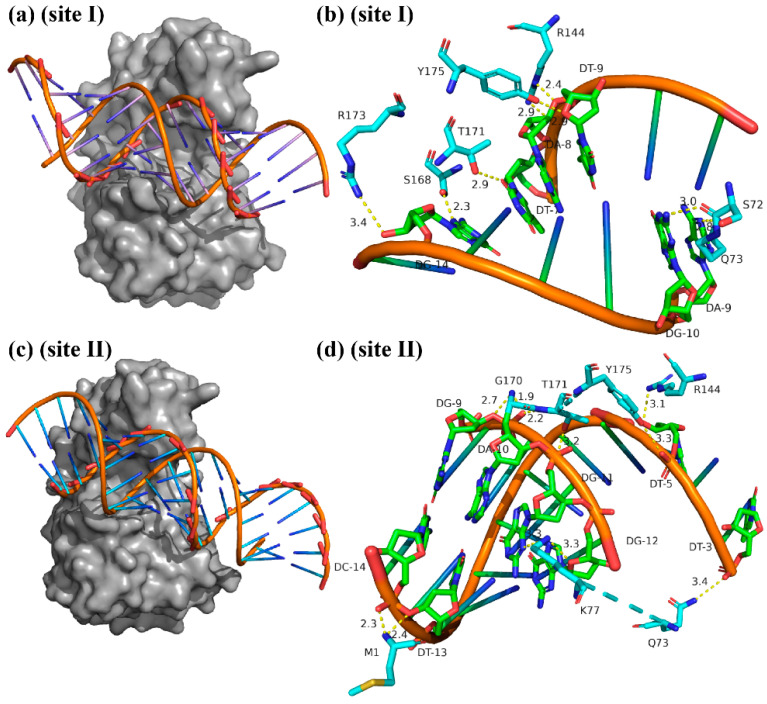
Molecular models of RRP_6_ protein interaction with site I (**a**,**b**) and site II (**c**,**d**).

The structural model of HPK_6_ and RRP_6_ proteins and their Ramachandran plots are shown in Figures S7 and S8, respectively. The Ramachandran plot showed that 100% of residues fell within the allowed regions, exceeding the 95% threshold considered acceptable for a high-quality model. This demonstrated that the structural model was appropriate for molecular docking and molecular dynamics simulations. The static potential energy diagrams of HPK_6_ and RRP_6_ proteins are shown in Figure S9. After 100 successful docking simulations were conducted, the optimal model was selected from the conformational results (Figure 4a,c and Figure S10). It was observed that a groove was present on the surface of the RRP_6_ protein, allowing DNA sequences (site I and II) to interact with these grooves. Additionally, it was noted that RRP_6_ protein could bind to the major groove of site I and the minor groove of site II. These results indicated that the groove on the surface of RRP_6_ protein played a crucial role in the binding process between RRP_6_ protein and DNA.

For site I, RRP_6_ protein interacted with a significant region of DNA within an AT-rich area, surrounded by base pairs T9, A8, A9, G10, and G14 (Figure 4b). For site II, RRP_6_ protein bound to the DNA region rich in AT bases, and the bases G9, A10, G12, T3, T5 and T13 played a key role in the binding (Figure 4d). Similarly, different amino acids in the RRP_6_ protein bound to two different sites. The amino acids playing the key role in the binding were S72, Q73, R144, S168, T171, R173, and Y175 for site I; M1, Q73, K77, R144, G170, T171, and Y175 for site II. Analysis of amino acids interacting with DNA sequence at the two binding sites revealed that Q73, R144, T171 and Y175 were present in both models. These amino acids not only possessed functional side chain groups but also were located in critical regions of the grooves of RRP_6_ protein. These results suggested that amino acids Q73, R144, T171 and Y175 played a leading role in facilitating the binding process between RRP_6_ protein and DNA sequence.

### 3.6. Mutated Protein and Nucleic Acid Interaction Detection

Amino acids located near the binding pocket of the RRP_6_ protein for DNA binding were analyzed, revealing that Q73, R144, T171, and Y175 were important amino acids (Figure 4b,d). Alanine-scanning mutagenesis was separately performed on these four amino acids. The results indicated that mutation to alanine significantly reduced the binding capability and stability of the protein. After mutating Q73, R144, T171 and Y175 into alanine, the energy values (∆G) of the proteins of RRP_6_-∆Q73, RRP_6_-∆R144, RRP_6_-∆T171, and RRP_6_-∆Y175 decreased to −0.87102177, −0.64210328, −0.68420012, and −1.6993903 respectively. These results further demonstrated that these amino acids played essential roles in the in vitro binding process of RRP_6_ protein.

**Figure 5:**
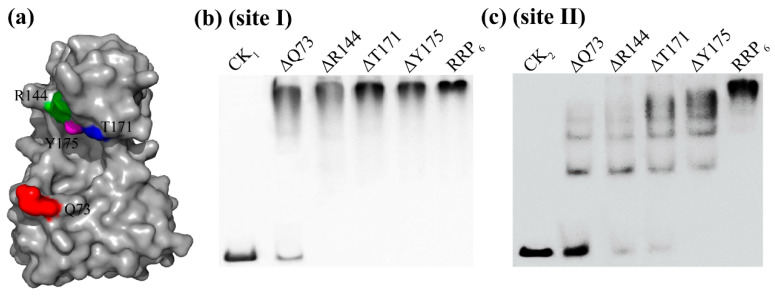
Site-specific mutation of key amino acids and the EMSA of mutant proteins with sites I and II. (**a**): Selection of mutation sites; (**b**,**c**): EMSA of mutant proteins with sites I and II; CK_1_ and CK_2_ represent the nucleic acids at site I and site II, respectively.

Based on Figure 5a, the binding model resembled a clip structure, with ΔQ73 located at the bottom playing a crucial supporting role. The four mutant proteins were successfully purified and their band sizes closely resembled those of the RRP_6_ protein (Figure S11). EMSA analysis revealed that during the binding process of the four mutant protein samples to sites I and II, the band migration distances were all larger than those of the RRP_6_ sample, and the ΔQ73 sample had the largest migration distance (Figure 5b,c). This indicated that after Q73 was mutated to alanine, the binding interaction between the protein and the nucleic acid (sites I and II) was significantly weakened, with some unbound probes remaining. In addition, compared with the control group, the band migration distances of samples ΔR144, ΔT171, and ΔY175 gradually decreased and were even smaller than those of the ΔQ73 sample (Figure 5b). These results suggested that while these amino acids could still bind to sites I and II after mutating to alanine respectively, their binding abilities were all weakened. Moreover, the ΔQ73 sample had the weakest binding ability to sites I and II, indicating that these four amino acids play important roles in the binding process of the RRP_6_ protein to sites I and II, and Q73 plays a key role in this binding process.

### 3.7. Effects of rrp_6_ Gene Knockout on the Growth of L. plantarum 163 and the Production of Plantaricin EF

**Figure 6:**
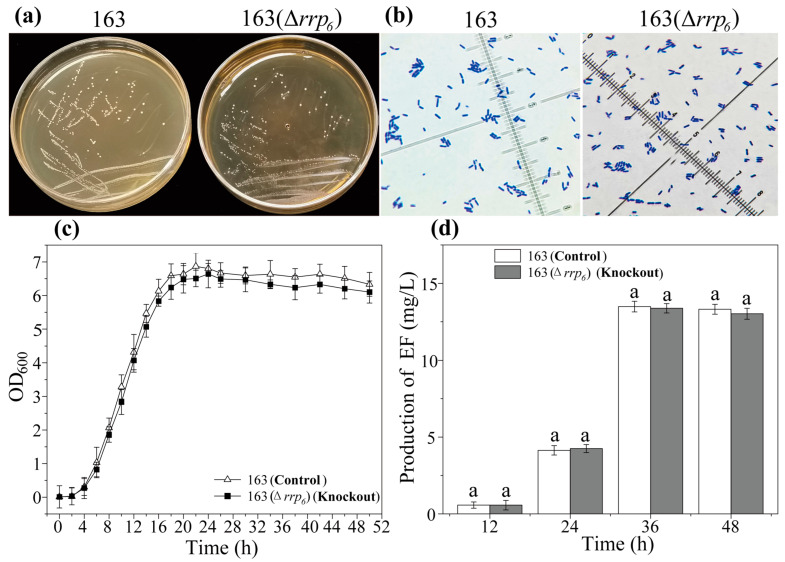
Effects of *rrp_6_* gene knockout in *L. plantarum* 163 on growth and plantaricin EF production. (**a**): Growth status; (**b**): Gram staining; (**c**): Growth curves; (**d**): Plantaricin EF production. 163: *L. plantarum* 163; 163(Δ*rrp_6_*): *rrp_6_* gene knockout in *L. plantarum* 163. The same lowercase letters indicate that there is no statistically significant difference within the same group (*p* > 0.05). Mean ± SD, *n* = 3, technical replicates.

Based on Figure S12, electrophoresis analysis indicated that no amplification bands were detected in the reaction system when PCR amplification of the target strain was conducted using specific primers for the *rrp_6_* gene. In stark contrast, the wild-type strain exhibited a distinct and single band under identical primer and amplification conditions, with a fragment size of 606 bp. This finding suggested that the *rrp_6_* gene in the target strain was successfully knocked out. The deletion of *rrp_6_* did not alter the basic cellular morphology or growth of *L. plantarum* 163. The knockout strain exhibited a cellular morphology similar to that of the wild-type (Figure 6a,b), as well as a growth curve indistinguishable from the wild-type (Figure 6c and Figure S13). In combination with Figure 1, neither the overexpression nor the knockout of the *rrp_6_* gene resulted in any changes in the basic cellular morphology or growth. Surprisingly, the deletion of *rrp_6_* also did not affect plantaricin EF biosynthesis. The production profile over time and final yield of plantaricin EF in the *L. plantarum* 163(Δ*rrp_6_*) were not significantly different from those of the wild-type strain at any time point measured (*p* > 0.05) (Figure 6d and Figure S14).

These results indicated that the knockout of the *rrp_6_* gene neither had an adverse effect on the growth of *L. plantarum* 163, nor inhibited the biosynthesis of plantaricin EF.

### 3.8. Effects of rrp_6_ Gene Knockout on the Transcription Levels of pln Locus Associated Genes in L. plantarum 163

**Figure 7:**
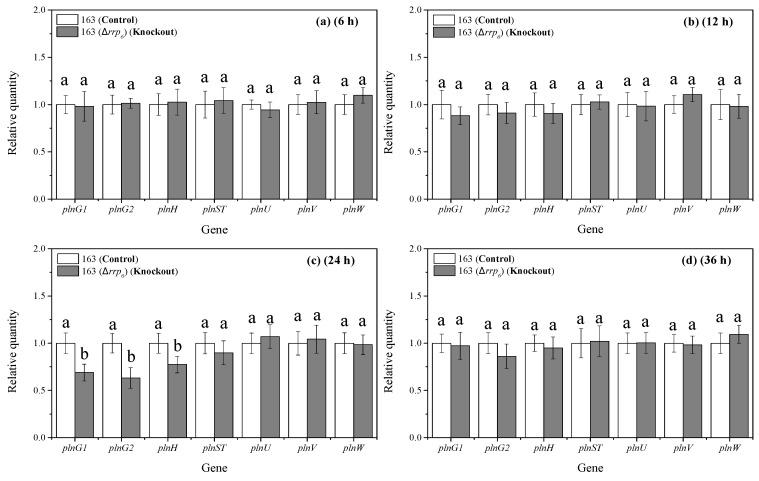
Effects of *rrp_6_* gene knockout on the related genes of the *pln* locus. (**a**) Transcription levels at 6 h; (**b**) Transcription levels at 12 h; (**c**) Transcription levels at 24 h; (**d**) Transcription levels at 36 h. 163: *L. plantarum* 163; 163(Δ*rrp_6_*): *rrp_6_* gene knockout in *L. plantarum* 163. The different lowercase letters indicate significant difference in the same group (*p* < 0.05). Mean ± SD, *n* = 6, technical replicates.

As shown in Figure 7, at 12 h, the relative transcription levels of the *plnG1*, *plnG2*, *plnH*, *plnST*, *plnU*, *plnV* and *plnW* genes in both the control and the knockout groups remained stable without significant fluctuations. Furthermore, the relative transcriptional levels between the two groups were similar. At 24 h, however, the relative transcriptional levels of the *plnG1*, *plnG2*, and *plnH* genes in the knockout group significantly decreased compared to those in the control group, by approximately 31%, 37%, and 23% respectively (*p* < 0.05). No significant differences were noted in the relative transcriptional levels of other genes between the two groups. At 36 h, the relative transcriptional levels of the three aforementioned genes in the knockout group increased and were nearly equivalent to those of the control group. The decline in transcriptional levels of these three genes within the knockout group suggested that there was an association between the HPK_6_/RRP_6_ and these specific genes located in the gene cluster *pln* locus. This implied that RRP_6_ could enhance the transcription and expression of these genes, thereby playing a crucial role in promoting plantaricin EF biosynthesis.

## 4. Discussion

The genomic data suggested that HPK_6_/RRP_6_ might be associated with the plantaricin biosynthesis in *L. plantarum* 163 (Figures S1 and S2). The overexpression of regulatory factor RRP_6_ resulted in an elevated transcription level of related genes (Figure 2). Meanwhile, it significantly enhanced the production of plantaricin EF (Figure 1). These results demonstrated that HPK_6_/RRP_6_ was not only implicated in the biosynthesis of plantaricin EF but also played a crucial role in promoting its production. It has been reported that the biosynthesis of bacteriocin is not only regulated by its own QSS but also by the TCS [20]. For instance, the biosynthesis of plantarcin Q7 can be indirectly regulated by the TCS AgrA/AgrC in *Lactiplantibacillus plantarum* Q7 [32]. In *Streptococcus gordonii*, the upregulation of the CiaRH TCS inhibited the Com pathway, thereby suppressing the biosynthesis of bactericin [33,34,35]. Therefore, investigating the regulatory mechanism of the HPK_6_/RRP_6_ on the biosynthesis of plantaricin EF can contribute to increasing its yield.

In the TCS HPK_6_/RRP_6_, HPK_6_ is a histidine protein kinase, and RRP_6_ serves as its homologous regulatory factor, which is a member of the LuxR family (Figure S15). The structure of this type of regulatory factor exhibits distinct characteristics. The N-terminal region serves as the signal-sensing domain, while the C-terminal region comprises the DNA-binding domain that contains the helical-turn-helical (HTH) motif. Moreover, the N-terminus often includes either an autoinducer binding domain or a response regulatory domain [36,37,38]. Research has demonstrated that LuxR-type HTH regulators play a crucial role in various biological processes and are involved in mediating diverse biological functions [39,40,41]. In our study, the RRP_6_ regulatory factor from the LuxR family could significantly enhance the transcriptional levels of related genes in the *pln* locus, thereby promoting the biosynthesis of plantaricin EF. Sequence analysis revealed that RRP_6_ protein was highly similar to the LuxR-type regulatory factors reported in *L. plantarum* WP 071542686.1 and *L. plantarum* WP 085439356.1. Moreover, such regulatory factors are widely distributed in *L. plantarum* (Figures S16–S18).

Many studies have reported on the regulatory factors associated with the LuxR family, and most members of this family play a positive regulatory role. For instance, as a transcriptional activator belonging to the LuxR family, MalT is capable of binding both ATP and maltotriose. By recognizing the MalT box sequence on DNA, it plays a positive regulatory role in the transcription of genes associated with maltose transport and metabolism in *Escherichia coli* [42]. Furthermore, in *Bacillus* species, GerE functions as an autonomic effector within the LuxR family; it binds to DNA through its HTH motif, positively regulates the expression of coat protein genes in spores, enhances spore maturation, and imparts resistance characteristics to them [36].

In this study, the overexpression of LuxR-type regulator (RRP_6_) could significantly enhance the transcriptional levels of related genes encoding the ABC transporter system (*plnG1*, *plnG2*, *plnH*) while not affecting the strain growth. Meanwhile, it could promote the biosynthesis of plantaricin EF in *L. plantarum* (Figure 1 and Figure 2). The regulatory mechanism indicated that RRP_6_ protein could bind to the two specific binding sites (Site I: ACGTTAATAGATAGTTGGC, 5′-3′, 19 bp; Site II: TAATTGGTGAGGGGAGTACAAG, 5′-3′, 22 bp) of the *plnG1* promoter sequence (Figure 3). Moreover, RRP_6_ protein bound to the major groove at site I and the minor groove at site II, with Q73 playing the key role in its binding process. The regulatory mode of RRP_6_ is similar to that of the regulatory factor Lp_2642. The Lp_2642 protein can bind to the major groove of the promoter sequence of the AIP gene *plnA*, thereby promoting its transcription and further enhancing the yield of plantaricin [20]. In addition, the knockout of the *rrp_6_* gene did not affect cell growth. Moreover, the production of plantaricin EF showed no significant difference compared with that of the wild-type strain (Figure 6). However, at 24 h, the transcriptional levels of *plnG1*, *plnG2*, and *plnH* genes in the knockout strain exhibited a significant decrease, by about 31%, 37%, and 23% respectively. These results may indicate potential regulatory compensation in the genome of *L. plantarum* 163, which compensates for the loss following the deletion of *rrp_6_*. This potential regulatory compensation mechanism will be the target of our next research. 

It is well known that bacteria use the TCS to sense changes in the environment and adjust metabolic behavior [43]. The system consists of protein kinase (PK) and response regulator (RR). Extracellular environmental signaling factors can phosphorylate PK on the cell membrane [44,45]. The phosphorylated PK then activates its homologous RR within the cell, and the activated RR further exerts its regulatory functions [46,47]. In this study, the auto-inducer of the histidine protein kinase (HPK) HPK_6_ in the TCS remains unclear. This is because the activation of HPK_6_ may be influenced by various factors in the environment, including salt ions and small-molecule metabolites. For example, it was reported that acetate ions could also activate the PlnB (HPK) in the TCS, thereby promoting the biosynthesis of plantaricin [48]. Therefore, the auto-inducers of the HPK_6_/RRP_6_ will be further investigated in the future.

During the growth of *L. plantarum* 163, the regulatory program is activated when the concentration of the extracellular AIP (PlnA) reaches a specific threshold. Upon binding to the PlnB (HPK) located on the cell membrane, PlnA can stimulate its HPK activity, leading to ATP hydrolysis and subsequently transferring a phosphate group to the regulatory factor PlnD. The phosphorylated PlnD then binds to the promoter regions of both PlnA and plantaricin synthesis genes, thereby initiating the biosynthesis of precursors of PlnA and plantaricin. As these precursors are transported from within the cell to the external environment, their signal peptides are cleaved by a specialized ABC transport system. Ultimately, mature forms of PlnA and plantaricin are secreted into the extracellular space via this ABC transport system, where they exert their biological functions [15,49]. During this process, the expression of the regulatory factor RRP_6_ enhances both the transcription and translation of genes associated with the ABC transport system. Consequently, it accelerates the secretion of PlnA and plantaricin EF, resulting in reduced intracellular and increased extracellular concentrations of them. The extracellular PlnA will continue to activate the PlnB, promoting the transcription and translation of intracellular PlnA and plantaricin EF, and forming a positive feedback loop.

## 5. Conclusions

In summary, through previous multi-omics joint analysis, the TCS HPK_6_/RRP_6_ related to the *pln* locus gene cluster of the plantaricin synthesis gene was screened out. Overexpression of the regulatory factor RRP_6_ increased the transcriptional levels of genes related to plantaricin and AIP transport and secretion in the *pln* locus. This further increased the extracellular concentrations of plantaricin and AIP. The potential regulatory mechanisms have shown that the RRP_6_ protein can specifically bind to the promoter sequence of the transporter-encoding gene *plnG1*, enhance its transcription and translation, and accelerate the transport and secretion of plantaricin and AIP, and thereby increase the yield of plantaricin EF. These results offer a novel strategy for achieving high-level production of plantaricin EF and contribute significantly to its large-scale application as a natural and safe food preservative in agriculture and the food industry.

## Figures and Tables

**Table 1:** Gene function of *pln* locus in *L. plantarum* 163 genome.

Gene Name	Function	Gene Name	Function
*plnorf3*	Bacteriocin	*plnE*	Bacteriocin
*plnorf5*	Immunoprotein	*plnG1*	ABC transporter protein
*plnorfZ3*	Unknown function	*plnG2*	ABC transporter protein
*plnR*	Unknown function	*plnH*	ABC transporter, accessory factor
*plnL*	Immunoprotein	*plnST*	Integral membrane protein
*plnK*	Bacteriocin	*plnU*	Integral membrane protein
*plnA*	Self-induced peptide	*plnV*	Integral membrane protein
*plnB*	Histidine protein kinase	*plnW*	Integral membrane protein
*plnD*	Response regulator	*plnX*	Type II toxin-antitoxin system RelE/ParE family toxin
*plnI*	Immunoprotein	*plnY*	HigA family addiction module antitoxin
*plnF*	Bacteriocin		

## Data Availability

The original contributions presented in this study are included in the article/Supplementary Materials, and further inquiries can be directed to the corresponding author.

## Supplementary Materials

The following supporting information can be downloaded at: https://www.mdpi.com/article/10.3390/microorganisms13122780/s1, Figure S1: The genome, transcriptome and proteome data were analyzed using the BGI Cloud platform to identify the proteins that interact with the *pln* locus; Figure S2: String network of protein–protein interaction to identify the proteins that interact with the *pln* locus; Figure S3: Effects of *rrp_6_* overexpression on the growth of *L. plantarum* 163; Figure S4: RP-HPLC analysis of plantaricin E and F by 163(*rrp_6_*); Figure S5: SDS-PAGE results of RRP_6_ protein; Figure S6: SDS-PAGE after RRP_6_ purification; Figure S7: Homologous modeling of HPK_6_ and RRP_6_; Figure S8: Ramchndran plot prediction of HPK_6_ and RRP_6_; Figure S9: The static potential energy diagram of HPK_6_ and RRP_6_; Figure S10: Molecular modeling of protein RRP_6_ interaction with two binding sites; Figure S11: SDS-PAGE after mutant protein purification; Figure S12: PCR verification of gene *rrp_6_* knockout strains; Figure S13: Effects of *rrp_6_* knockout on the growth of *L. plantarum* 163; Figure S14: RP-HPLC analysis of plantaricin E and F by 163(Δ*rrp_6_*); Figure S15: The domain of the two-component systems HPK_6_/RRP_6_; Figure S16: Multiple sequence alignment of RRP_6_ homolog proteins; Figure S17: Conservation analysis of RRP_6_ homolog proteins; Figure S18: Phylogenetic tree of the amino acid sequence for the RRP_6_ protein; Table S1: Strains and plasmids presented in this study; Table S2: Primers used in this study; Table S3: qPCR primers used in this study.

## Abbreviations

The following abbreviations are used in this manuscript:

AIP	Auto-inducing peptide
LAB	Lactic acid bacteria
QSS	Quorum-sensing system
TCS	Two-component system
CFS	Cell-free supernatant
EMSA	Electrophoretic mobility shift assay
PK	Protein kinase
RR	Response regulator
HPK	Histidine protein kinase
