# Supplementary material for: LuxR-Type Regulator RRP6 Positively Regulates the Biosynthesis of Plantaricin EF and Improves Its Production in Lactiplantibacillus plantarum 163
Source: Microorganisms. 2025 Dec 6;13(12):2780. doi: 10.3390/microorganisms13122780 (PMC12735751; doi:10.3390/microorganisms13122780)
Supplement: Supplementary file 1 [file microorganisms-13-02780-s001.zip › microorganisms-4001710-supplementary.pdf]

Supplementary materials

LuxR-Type Regulator RRP_6 Positively Regulates the Biosynthesis of Plantaricin EF and Improves Its Production in *Lactiplantibacillus plantarum* 163

Yaxuan Liu, Siqi Liu, Zixian Li, Chuangen Huo, Guangli Wang, Xin Zeng, Bingyue Xin and Deyin Zhao *

School of Life Science, Huaibei Normal University, Huaibei 235000, China

* Correspondence: zhaody@chnu.edu.cn

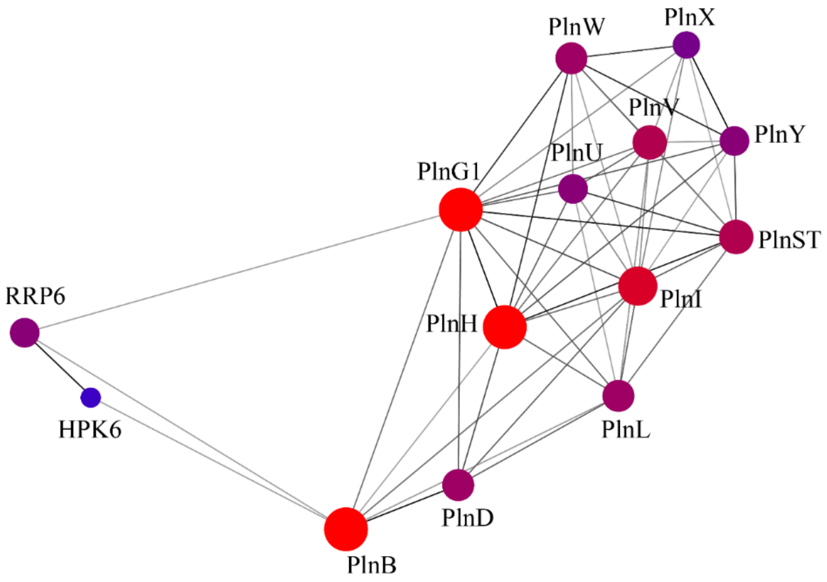

Figure S1 The genome, transcriptome and proteome data were analyzed using the BGI Cloud platform to identify the proteins that interact with the *pln* locus

Note: The color from purple to red indicates increasing interaction strength, and the thicker the line, the stronger interaction.

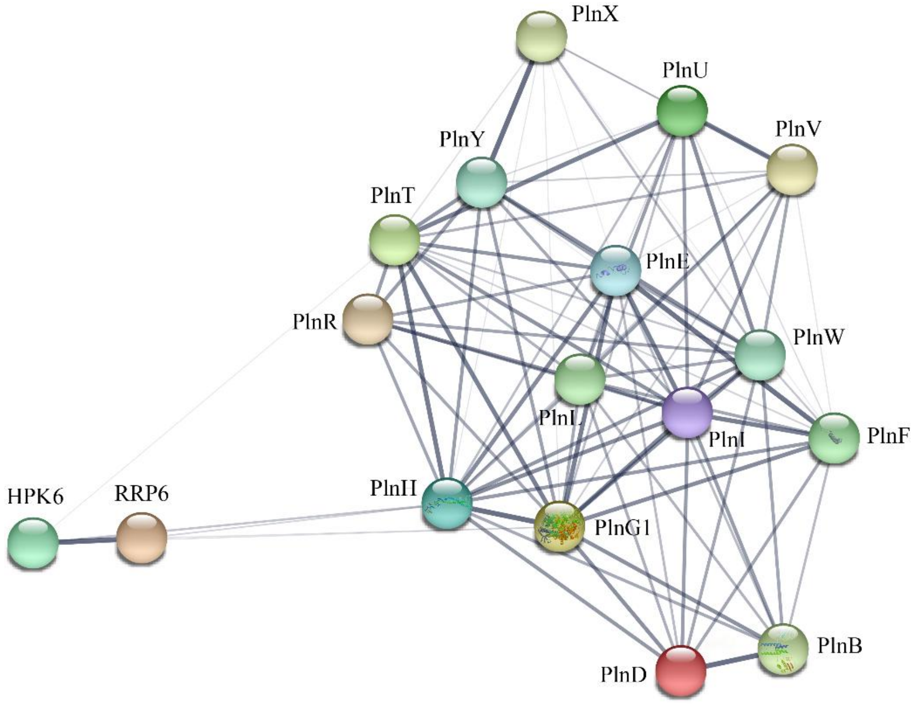

Figure S2 String network of protein-protein interaction to identify the proteins that interact with the *pln* locus

Note: The thicker the line indicates the stronger interaction.

Gene sequence of HPK_6 and RRP_6:

1. **HPK_6 (1110 bp)**

ATGGCGTTGTTGACGCATCATAAAAATAAATTGAAGACGATTGAATGGACGAGCTACGT
GTGGCTCGCCTATTTGCCGTATACAATTGCGGCTTACATACCCGTTAAATCTTGGCATGA
TTGGCTTTGGCTATTGCTTGTTGGTGGTTTTCTTGTGTTGTACATCTTGGTTGTCGAGAA
GCCACATTGGCGTGCAGTGACGATGCCAGCGGAGTTAGTTGTGACGGGACTGTTTGCG
GTATTTGCGGCTAACAATTTTATGATCATCTTTCCCGGCTGGCAAGTGAGCTTTTTACTT
GGTCGTCGTCCTAAACGAGAATTTTATGGTTTTGCTTTAGGGTATTATGCCTTTTTAATT
GGTGGCTGTATACGAGACTATTTACAATATCCTGGTGTGTTTGGGTGGCATAATGGTGAC
GTGATGGGCTTAGTTTTTCCATTGTTATCACCCATACTCGCGTATACCTTTTCGTGGTCA
GTTGAACGACAGCGCCAACTCAACCAAACTAACCGCCGATTAAAAACAATTGTGGAA
CGTAACGAGCGTGAACGAATCGCGCGTGACTTGCACGATACCCTTGGACAAAGCTTTT
CAATGATTACACTCAAAACGGAATTAGCTAAAAAATTACTCGTCAAAGCACCGGAGAA
AGTGGCACCAGAATTGGACGAAATCGAACAAACCAGTCGCCAAAATTTACAGTTAGTT
CGCGAAATCGTCAATAATTTGCATGAACAATCGCTCACAGAAGTCCTATTGGCCCAAAC
TCGTAATCTAGCAGCGGTTGGTGTCTGGACGACGACCACTGGTGAGACACAGGCAAC
CAAGTGGCCGACAACGGTCCAAAGTTGTTTTGCGGCGGTGTTAGTTGAAGCCATGACC
AATGTGATGCGCCACGCACACGCGCACGAGGTGCGGATTGATTTGTCGAGACGGCAA
AAATTTATCAGATTAAGCTGAAAGATGACGGTAAAGGTGGAACCTTGATTCGTCAGGG
CGCCAACGGGATTGCCGGGATGCGAACCCGCTTGCAAGCTAAACAGGGCACCTTTGCC
ATCACGAGTAGTCGGCGCGGCACCCAGTTGATCTTAACTTTACCAAAGGAGTAG(5′-3′)

2. **RRP_6 (606 bp)**

ATGATAACTTTATACTTAGCAGAAGACCAAAGCATGTTGAATTCTGCCTTGACCCAATT
ACTGGAATTAGAAGATGATCTACACGTGGTGGGTAGCGCAGTAGACGGCATAAACGCC
TGGCAGGAGTTGCAAGAACTGCAACCGGATGTCGCAATTCTGGATATCGAAATGCCAG
GGATAACCGGCTTGGATGTTGCTGATCTATTGGACAGTTCGCAGCTAGCCACGAAAGTA
ATAGTTTTGACGACTTTTGCGCAACGCCGTTATTTTGAACGAGCAGTTAAAGCCAATGT
CGCGGGATATTTGTTAAAAGATAGTCCGAGTGATGACCTAATTGATGCAATTCGAGCTG
TGATGACCGGCCGCACCATCTATGCGCCCGAGCTAGTCACGAATATGTTGTCAGCAGAC
AACAATCCATTGACCGAACGGGAACTAGCAGTTTTGGTCGAAGCTGAGAAGGGGTTG
CCCACGAAGACGATTGCGGCCAATTTATACTTGTCAGCGGGGACGACCCGTAATTACTT
ATCCGCCATTTTTAGCAAATTGGGGGTTCACAATCGATTAGAAGCGATTCGTGTGGCGA
AAGCTAATCAGTGGTTGTAA (5′-3′)

Amino acid sequence of HPK_6:

1. HPK_6 (369 AA)

MALLTHHKNKLKTIEWTSYVWLAYLPYTIAAYIPVKSWHDWLWLLLVGGFLVLYILVVEK
PHWRAVTMPAELVVTGLFAVFAANNFMIIFPGWQVSFLLGRRPKREFYGFALGYYAFLIGG
CIRDYLQYPGVFGWHNGDVMGLVFPLLSPILAYTFSWSVERQRQLNQTNRRLKTIVERNE
RERIARDLHDTLGQSFSMITLKTELAKKLLVKAPEKVAPELDEIEQTSRQNLQLVREIVNNL
HEQSLTEVLLAQTRNLAAVGVWTTTTGETQATKWPTTVQSCFAAVLVEAMTNVMRHAH
AHEVRIDFVETAKIYQIKLKDDGKGGTLIRQGANGIAGMRTRLQAKQGTFAITSSRRGTQL
ILTLPKE*

Amino acid sequence of RRP_6:

2. RRP_6 (201 AA)

MITLYLAEDQSMLNSALTQLLELEDDLHVVGSAVDGINAWQELQELQPDVAILDIEMPGIT
GLDVADLLDSSQLATKVIVLTTFAQRRYFERAVKANVAGYLLKDSPSDDLIDAIRAVMTGR
TIYAPELVTNMLSADNNPLTERELAVLVEAEKGLPTKTIAANLYLSAGTTRNYLSAIFSKLG
VHNRLEAIRVAKANQWL*

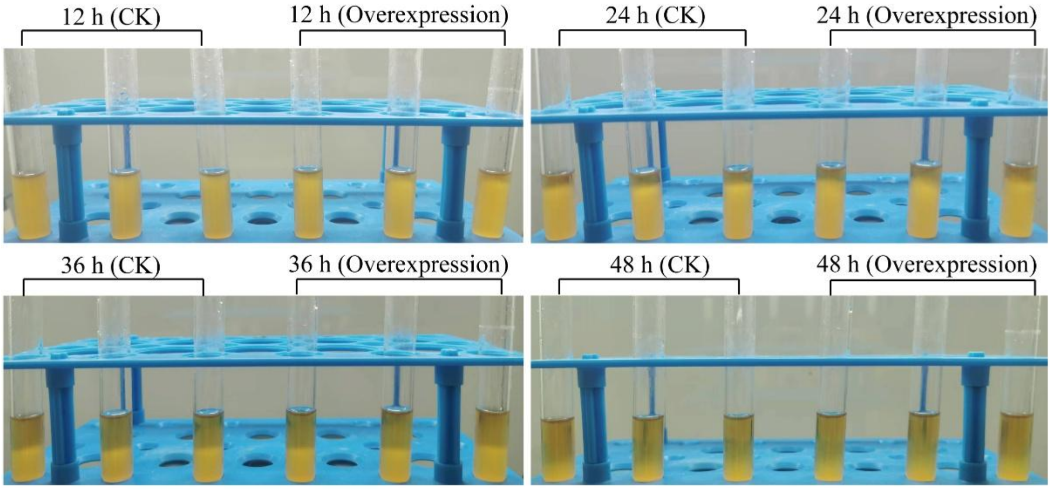

Figure S3 Effects of *rrp₆* overexpression on the growth of *L. plantarum* 163

Note: CK: *L. plantarum* 163; Overexpression: *rrp₆* gene overexpression in *L. plantarum* 163.

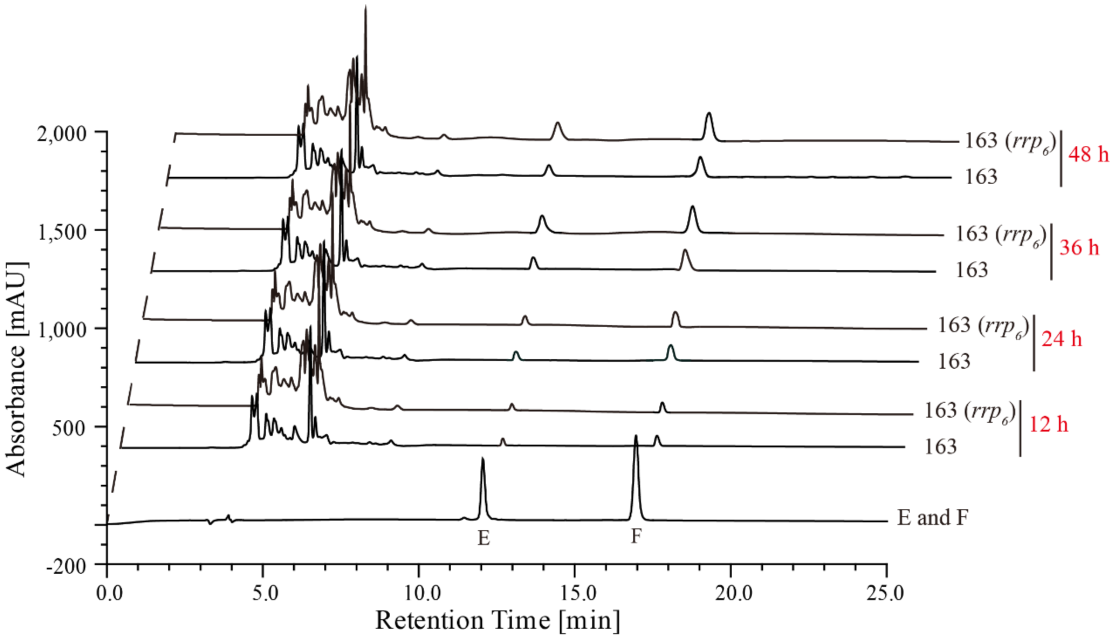

Figure S4 RP-HPLC analysis of plantaricin E and F produced by 163(*rrp₆*)

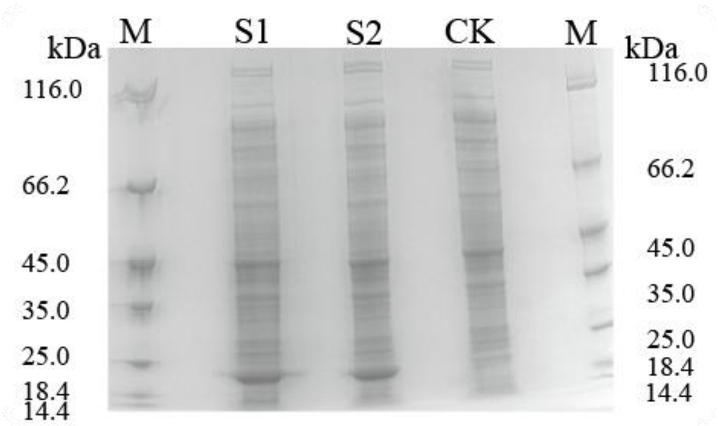

Figure S5 SDS-PAGE results of RRP_6 protein

Note: M: protein marker; CK: RRP_6 without induce; S1 and S2 are sample RRP6 after induced

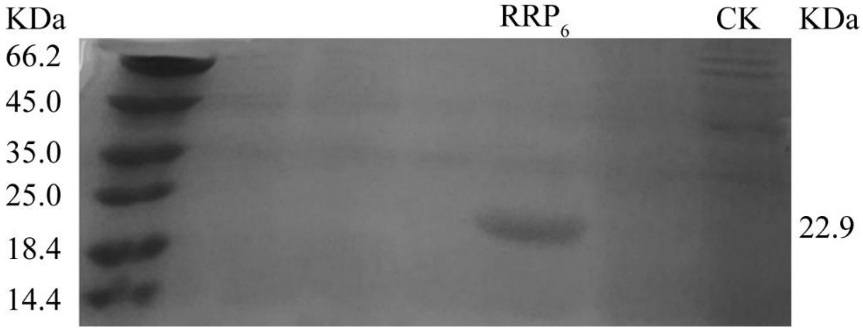

Figure S6 SDS-PAGE after RRP_6 purification

Note: M: protein marker; CK: RRP_6 without induce; RRP_6: purified protein of RRP_6.

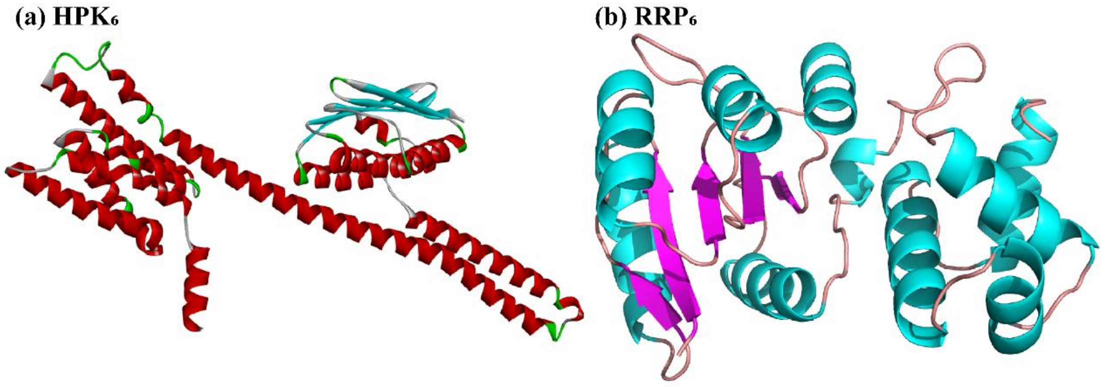

Figure S7 Homologous modeling of HPK_6 and RRP_6

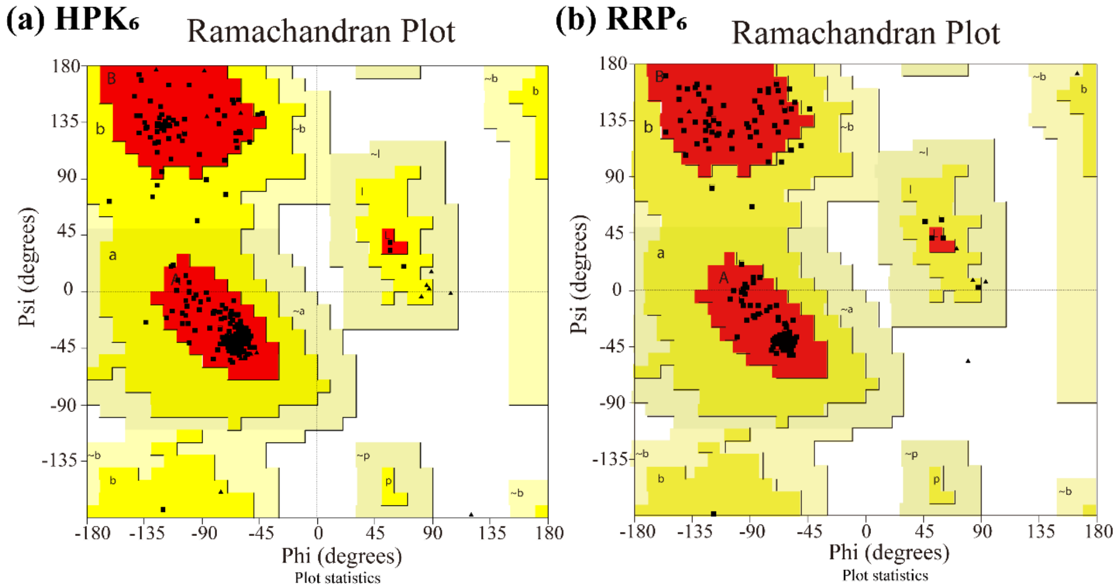

Plot statistics (a) HPK_6

Residues in most favoured regions [A,B,L]	318	96.4%
Residues in additional allowed regions [a,b,l,p]	12	36%
Residues in generously allowed regions [~a,~b,~l,~p]	0	0.0%
Residues in disallowed regions	0	0.0
	----	------
Number of non-glycine and non-proline residues	330	100.0%

Plot statistics (b) RRP_6

Residues in most favoured regions [A,B,L]	172	93.5%
Residues in additional allowed regions [a,b,l,p]	12	6.5%
Residues in generously allowed regions [~a,~b,~l,~p]	0	0.0%
Residues in disallowed regions	0	0.0%
	----	------
Number of non-glycine and non-proline residues	184	100.0%

Figure S8 Ramchndran plot prediction of HPK_6 and RRP_6

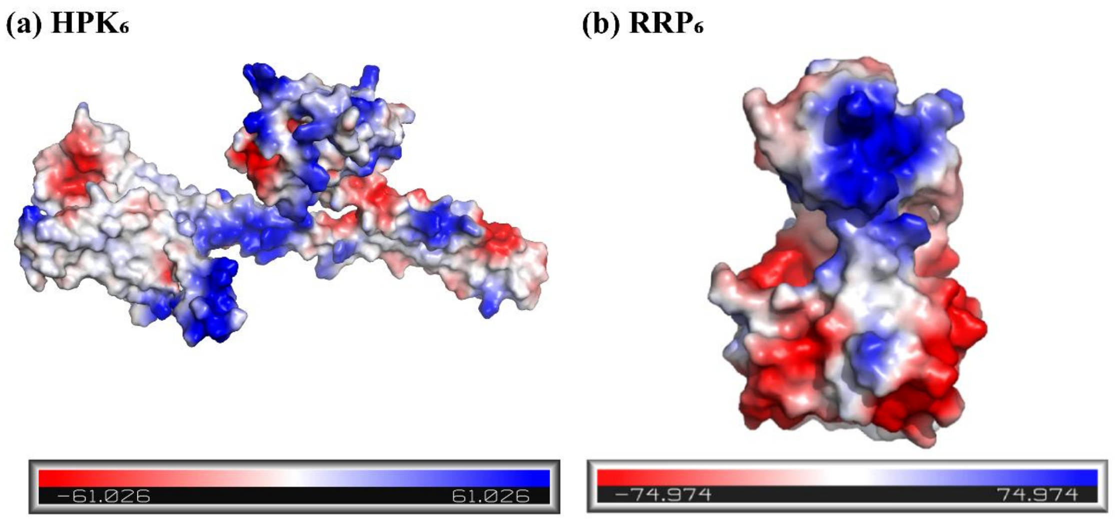

Figure S9 The static potential energy diagram of HPK_6 and RRP_6

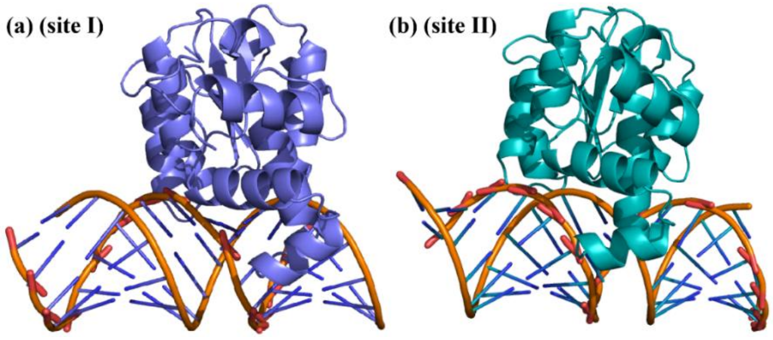

Figure S10 Molecular modeling of protein RRP_6 interaction with two binding sites.

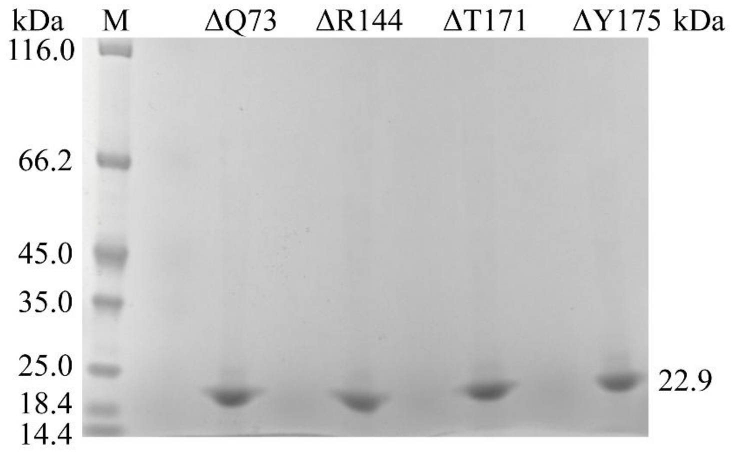

Figure S11 SDS-PAGE after mutant protein purification

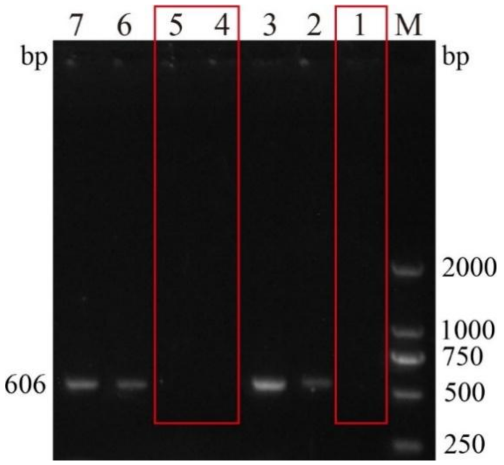

Figure S12 PCR verification of gene *rrp*$_6$ knockout strains

Note: M: DS2000 DNA marker; The size of the gene *rrp*$_6$ was 606 bp. PCR amplification was performed using the primers of the gene *rrp*$_6$. The lanes (1, 4 and 5) where bands could not be amplified were the mutant strains of the gene *rrp*$_6$ knockout.

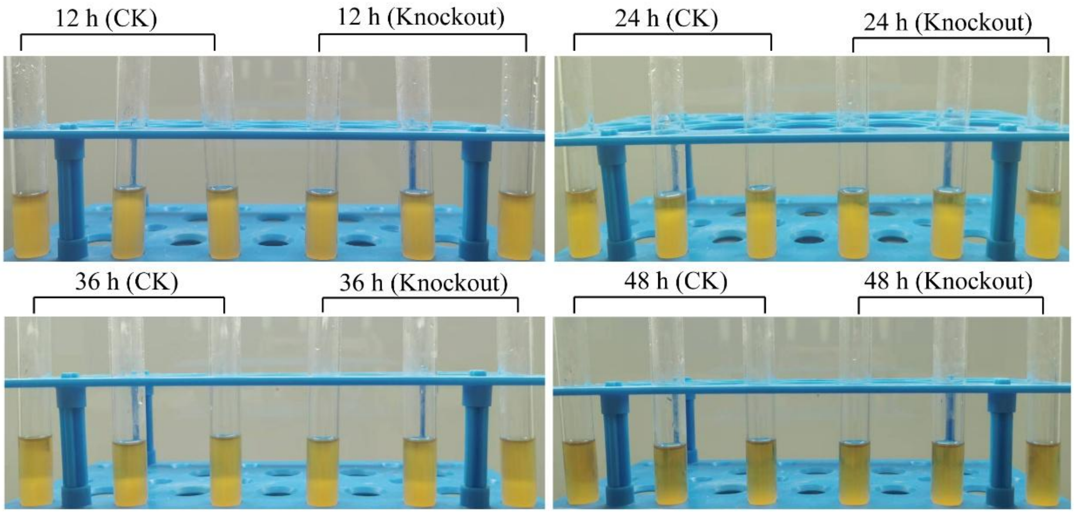

Figure S13 Effects of *rrp₆* knockout on the growth of *L. plantarum* 163

Note: CK: *L. plantarum* 163; Knockout: *rrp₆* gene knockout in *L. plantarum* 163.

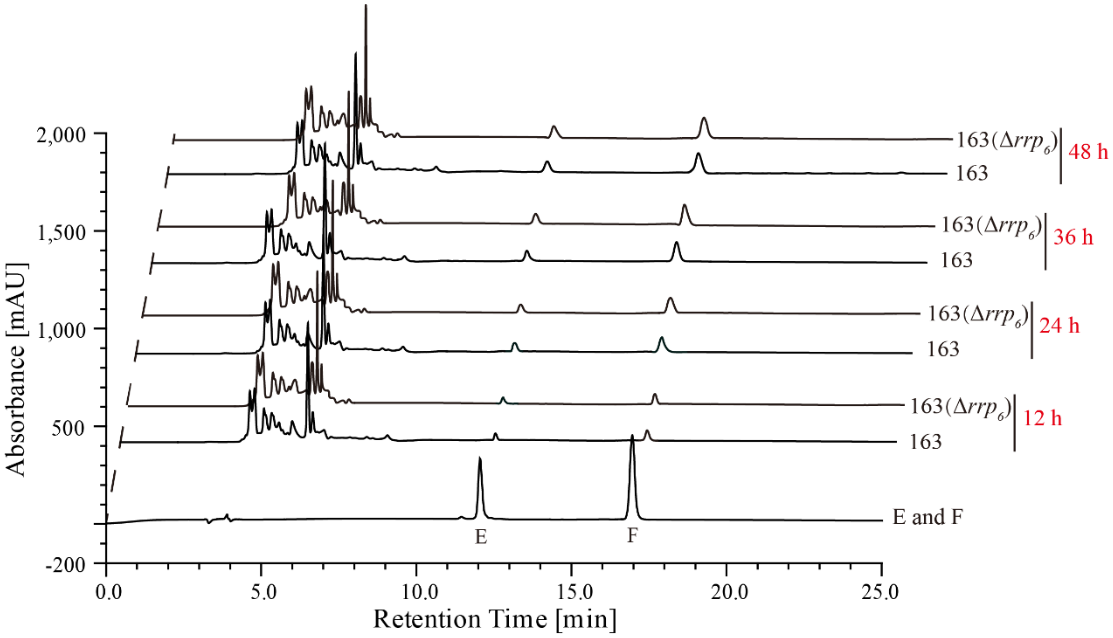

Figure S14 RP-HPLC analysis of plantaricin E and F produced by 163(Δ*rrp₆*)

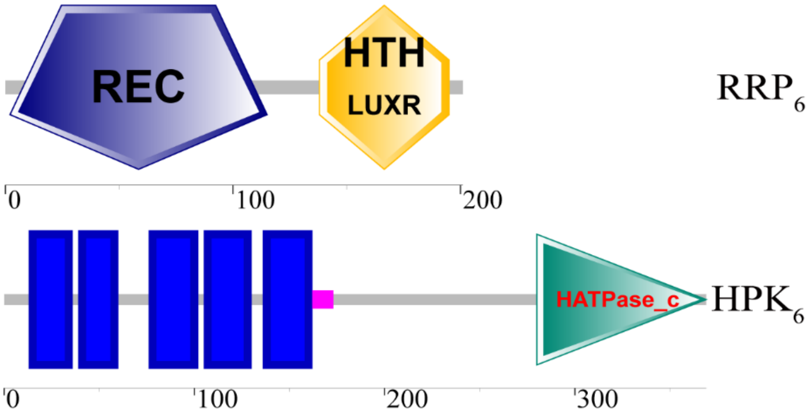

Figure S15 The domain of the two-component systems HPK_6/RRP_6.

Note：REC: Start from 2 to 115; HTH_LUXR: Start from 138 to 195; Transmembrane region 1: Start from 15 to 34; Transmembrane region 2: Start from 41 to 58; Transmembrane region 3: Start from 78 to 100; Transmembrane region 4: Start from 107 to 128; Transmembrane region 5: Start from 138 to 160; Low complexity: Start from 162 to 173; HATPase_c: Start from 280 to 369

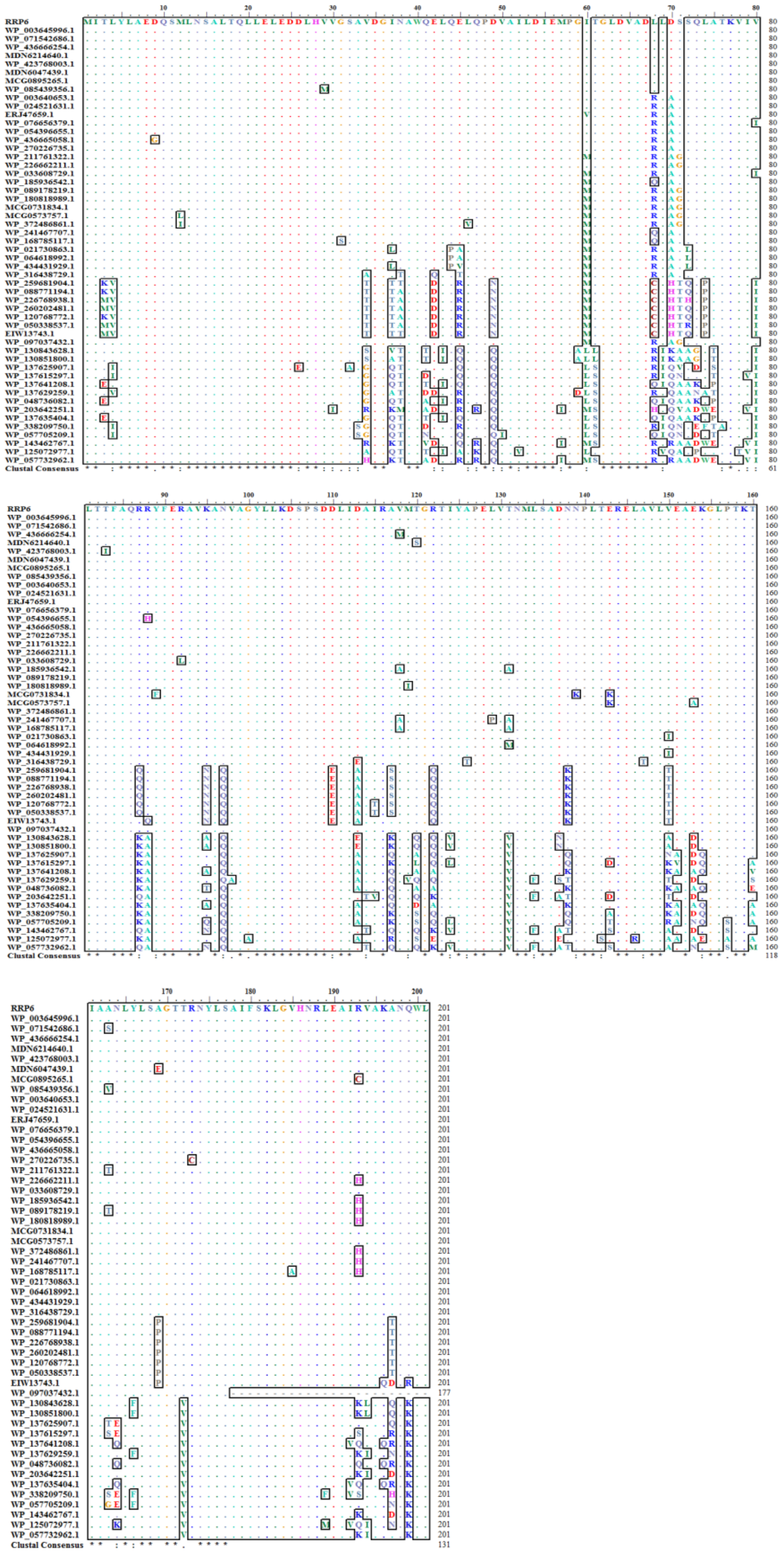

Figure S16 Multiple sequence alignment of RRP$_6$ homolog proteins

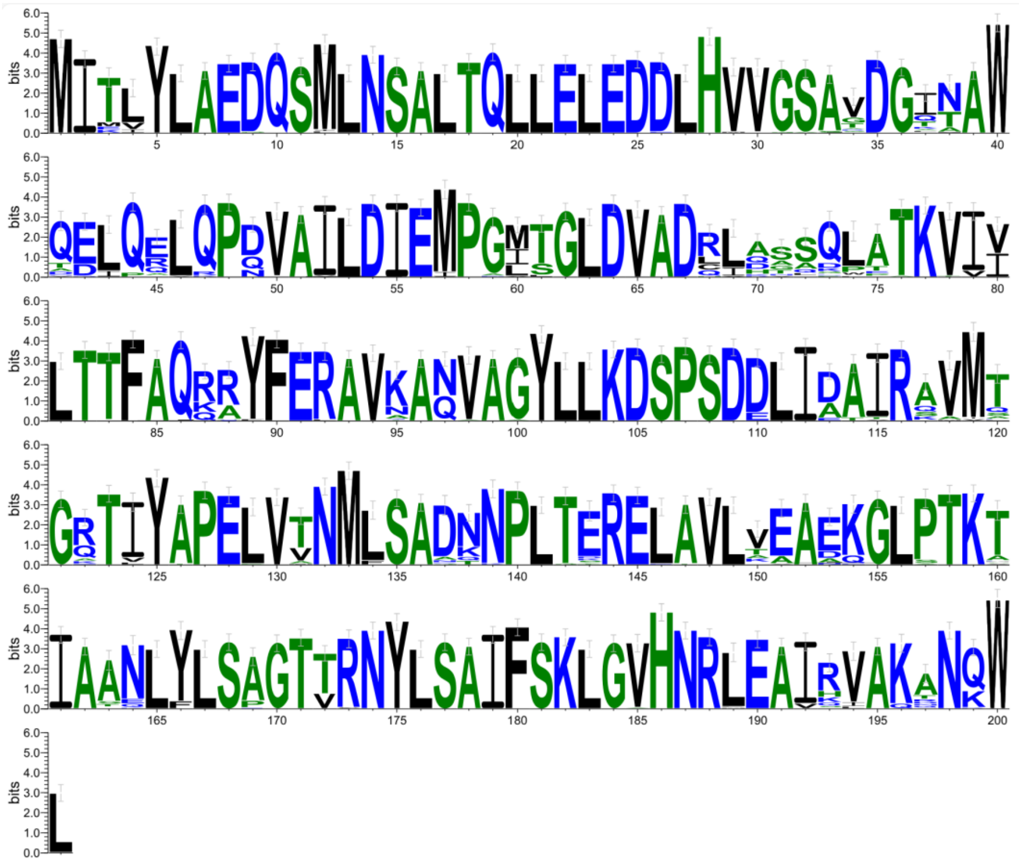

Figure S17 Conservation analysis of RRP_6 homolog proteins

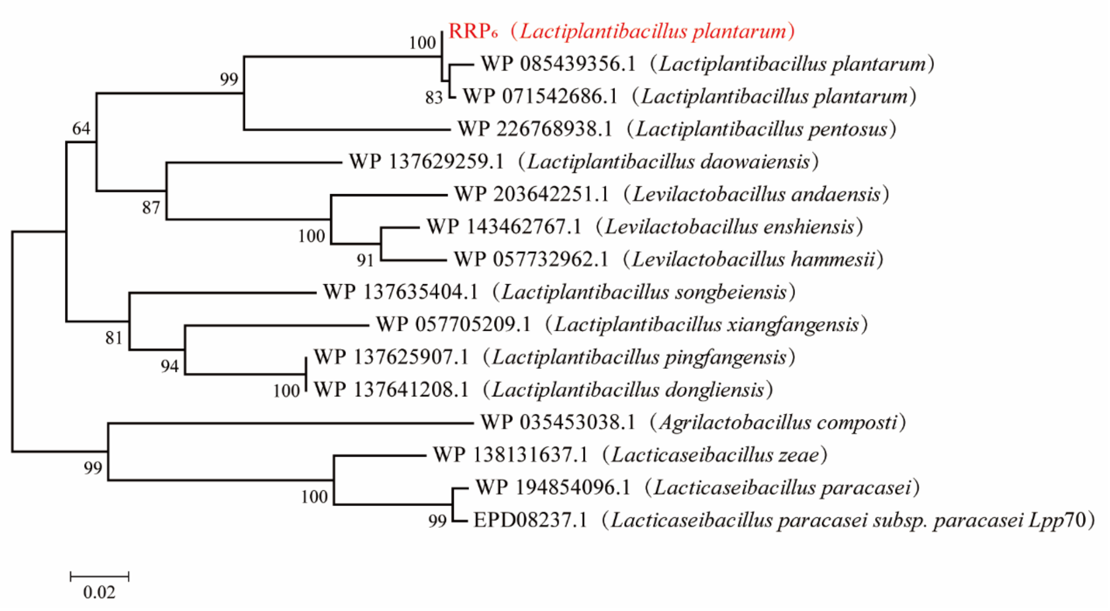

Figure S18 Phylogenetic tree of the amino acid sequence for the RRP_6 protein

Table S1 Strains and plasmids presented in this study

Strains or plasmids	Characteristics	Source
Plasmids		
pET30a	T7 promoter, lac operator, Kan^r	Novagen
pET30a-*rrp_6*	pET30a with *rrp_6* gene, Kan^r	This study
pMG36e	*E. coli* and *L. plantarum* shuttle, Em^r	Our lab
pMG36e-*rrp_6*	pMG36e with *rrp_6*, Em^r	This study
pNZ5319	*E. coli* and *L. plantarum* shuttle, Cm^r, Em^r	Our lab
pNZ5319-*rrp_6*	pNZ5319 with *rrp_6*, Cm^r, Em^r	This study
Strains		
E. coli DH5α	F-φ80 lac ZΔM15 Δ(lacZYA-arg F) U169 endA1 recA1 hsdR17(rk−,mk+) supE44λ- thi-1 gyrA96 relA1 phoA	TransGen Biotech
E. coli BL21(DE3)	F^- dcm ompT hsdS (rB^- mB^-) gal	Zoman Biotechnology, Beijing, China
E. coli BL21(DE3)(*rrp_6*)	F^- dcm ompT hsdS (rB^- mB^-) gal, pET30a with *rrp_6* gene, Kan^r	This study
L. plantarum 163	wild-type strain, producer of plantaricin EF	Our lab
L. plantarum 163(*rrp_6*)	*rrp_6* overexpression, derivative of strain 163, Em^r	This study

Kan^r: kanamycin resistance; Em^r: erythromycin resistance; Cm^r: chloramphenicol resistance

Table S2 Primers used in this study

Plasmid name	Primer name	Sequence 5′-3′
pMG36e	e-*rrp_6*F	agcagaaaaattcgtaattcATGATAACTTTATACTTAGCAGAAGACCAA
	e-*rrp_6*R	tccaaatatcgtagcgccggTTACAACCACTGATTAGCTTTCGC
	e-F	CCGGCGCTACGATATTTGG
	e-R	GAATTACGAATTTTTCTGCTGAAACG
pET30a	a-*rrp_6*F	aactttaagaaggagatataATGATAACTTTATACTTAGCAGAAGACCAA
	a-*rrp_6*R	agtgcggccgcaagcttgtcTTACAACCACTGATTAGCTTTCGC
	a-F	GACAAGCTTGCGGCCGCA
	a-R	TATATCTCCTTCTTAAAGTTAAACAAAATTATTT
pNZ5319	9-*rrp_6*F	attaaaggaccgataacgcgTTGGCTTTGGCTATTGCTTGT
	9-*rrp_6*R	tggcttctgtttctatcaggAACATTGATCCCCGCCAAC
	9-F	CCTGATAGAAACAGAAGCCACTGG
	9-R	CGCGTTATCGGTCCTTTAATTG

Table S3 qPCR primers used in this study

Gene name	Primer name	Sequence 5′-3′
16s rRNA	16s rRNA-F	GCATTAAGCATTCCGCCTGG
	16s rRNA-R	ACCTGTATCCATGTCCCCGA
plnG1	*plnG1*-F	AACGCATGTCGTGATTGCTG
	plnG1-R	CGGTGCAAAGAAAAGGGCAA
plnG2	*plnG2*-F	GACGAGTGGACTGGATGCAA
	plnG2-R	GACCCGACCATCATGCAGAA
plnH	*plnH*-F	CGAAGACCGCACAACAAGTG
	plnH-R	TGGCCTCCTTTGACTTAGCG
plnST	*plnST*-F	AAGCACCGCTTCCCATGATT
	plnST-R	ACGGCTCCCAATTTGCATCA
plnU	*plnU*-F	TGGTCAATGATGGGGCTGAC
	plnU-R	ACGGGGCTAACGATGTTTGT
plnV	*plnV*-F	TTTTGCACCGATTTGCGAGG
	plnV-R	ACTAAAACCGCCACGCAGTA
plnW	*plnW*-F	ATCGGCGCAGTCATTATCGT
	plnW-R	GGCATCGAGTGTCATCGCTA
